# Quorum Sensing Regulator CinR Directly Activates the Catalase–Peroxidase Gene *katG* to Alleviate Oxidative Stress and Promote Symbiotic Nitrogen Fixation in *Rhizobium etli* CFN42

**DOI:** 10.3390/antiox15060752

**Published:** 2026-06-15

**Authors:** Xuelian Chen, Tianyi Wu, Zhi Zheng, Chuling Gan, Jian Lin, Siqing Yin, Zi Li, Hongjian Liu, Yajun Cao, Zhi Huang, Hui Wang, Guoxi Zhang, Zengtao Zhong

**Affiliations:** 1 College of Life Sciences, Nanjing Agricultural University, Nanjing 210095, China; 2019216015@njau.edu.cn (X.C.); 2024216020@stu.njau.edu.cn (T.W.); 2020116075@stu.njau.edu.cn (Z.Z.); 2023116067@stu.njau.edu.cn (C.G.); 2022116055@stu.njau.edu.cn (J.L.); 15516976613@163.com (S.Y.); 2023116068@stu.njau.edu.cn (Z.L.); m13652062994@163.com (H.L.); caoyajun@njau.edu.cn (Y.C.); zhuang@njau.edu.cn (Z.H.); wanghui@njau.edu.cn (H.W.); 2 Nanjing Yuanjian Bioengineering Co., Ltd., Nanjing 210046, China

**Keywords:** *Rhizobium etli*, quorum sensing, oxidative stress, CinR, *katG*, symbiosis, nitrogen fixation

## Abstract

Many rhizobia use quorum sensing (QS) systems to detect their population density and modify their symbiotic behavior with the legume host. There are three LuxRI-type QS systems in *Rhizobium etli* CFN42, and CinR plays a key role in symbiotic performance. However, the details of how CinR regulates the symbiotic process remain unknown. In this study, we employed the RNA-Seq method to screen differentially expressed genes between the wild-type strain and the Δ*cinR* mutant of *R. etli* CFN42. We found that most of the genes related to reactive oxygen species (ROS) were expressed at lower levels in the Δ*cinR* mutant than in CFN42. We also found that the Δ*cinR* mutant was more sensitive to H_2_O_2_ than to CFN42. We then showed that CinR positively regulated *katG* expression and possessed an affinity to bind the *katG* promoter in the absence of the AHL ligand. The addition of AHLs promoted CinR binding to the *katG* promoter and enhanced *katG* expression. Accumulation of H_2_O_2_ and O_2_^•−^ was observed in root nodules formed by the Δ*cinR* mutant. Crucially, *katG* overexpression rescued the H_2_O_2_-sensitive phenotype in vitro and partially restored defective symbiotic performance in nodules formed by the Δ*cinR* mutant on the common bean. These results suggest that CinR globally regulates ROS scavenging gene expression in order to balance oxidative stress within root nodules, promoting nitrogenase activity of *R. etli* CFN42.

## 1. Introduction

Biological nitrogen fixation (BNF) is a cornerstone of the global nitrogen cycle and a key process for sustainable agriculture, which provides over 50 Tg of nitrogen to agriculture [1]. As the most efficient BNF system, symbiotic nitrogen fixation (SNF) requires rhizobia to exchange multiple signal transduction with their legume host. Many rhizobia employ *N*-acyl homoserine lactone (AHL) quorum sensing (QS) systems, which are synthesized by LuxI-type proteins and perceived by cognate LuxR-type transcriptional regulators [2], to optimize their symbiotic process with legume plants, including nodulation efficiency, biofilm formation, exopolysaccharide production and plasmid transfer [3,4,5,6]. *Rhizobium etli* CFN42, a microsymbiont of the common bean (*Phaseolus vulgaris* L.), possesses a complex QS network comprising at least three LuxRI-type systems: CinRI, RaiRI, and TraRI [7,8,9]. These systems are organized hierarchically, with the chromosome-encoded CinRI system globally regulating the expression and activity of the plasmid-encoded RaiRI and TraRI systems [8]. Our previous work demonstrated that the master regulator CinR plays a more important role in symbiotic performance than the other two regulators, RaiR and TraR [8]. However, how CinR regulates these symbiotic processes remains unknown.

Nitrogenase is highly sensitive to oxygen, being irreversibly inactivated by molecular oxygen and reactive oxygen species (ROS) [10]. To protect nitrogenase, legume nodules create a microaerobic environment through a combination of a physical oxygen diffusion barrier and the presence of oxygen-binding leghemoglobin [11]. Paradoxically, significant ROS are also produced while rhizobia interact with their host. During the early infection stage, a transient oxidative burst is triggered by rhizobial Nod factors. Throughout the functional lifespan of the nodule, superoxide anion (O_2_^•−^) and hydrogen peroxide (H_2_O_2_) are continuously generated as byproducts of highly active metabolic processes, including respiration and nitrogen fixation itself [12,13]. An over-accumulation of these ROS can lead to lipid peroxidation, protein damage, DNA mutation, and, ultimately, inactivation of nitrogenase and premature nodule senescence [12,14]. Therefore, successful symbiosis relies heavily on the bacteroid’s ability to maintain robust antioxidant defense systems to scavenge these harmful ROS molecules. The most important enzymes in ROS scavenging systems include superoxide dismutases (SODs), which convert O_2_^•−^ to H_2_O_2_, and catalases and peroxidases, which subsequently detoxify H_2_O_2_ to water and oxygen [15]. Nodules exhibit impaired symbiotic performance when these enzymes are absent [16]. Similarly, the bacterioferritin comigratory protein (BCP) has been shown to be critical for H_2_O_2_ resistance and nitrogen fixation in *Azorhizobium caulinodans* [17]. To keep the SNF running smoothly, rhizobia use a set of common regulators that exist in most bacteria to balance oxidative stress in nodules, such as OxyR and SoxR. Because thousands of bacteroids reside in a single nodule, the question arises as to whether a special regulator is involved in ROS responses by sensing population density.

To fully uncover the regulatory pathway of CinR in symbiosis, we performed a transcriptome analysis of nodules formed by wild-type and Δ*cinR* mutant strains, identifying potential targeting genes affected by CinR in nodules. We found that most of the genes involved in ROS scavenging were expressed at lower levels in nodules formed by the Δ*cinR* mutant than in those of CFN42. Interestingly, we found that CinR directly induced *katG* expression. The scenario in which CinR, the QS regulator, cooperates with OxyR to regulate catalase gene expression has not been observed in other bacteria. We further show that this CinR-*katG* regulatory axis is active in planta and is crucial for limiting H_2_O_2_ and O_2_^•−^ accumulation within the nodule and maintaining optimal symbiotic nitrogenase activity. Our findings reveal a novel mechanism by which a QS system directly influences the oxidative stress response of a symbiont to achieve successful mutualism.

## 2. Materials and Methods

### 2.1. Bacterial Strains and Growth Conditions

*Escherichia coli* strains were grown in Luria–Bertani (LB) medium at 37 °C. *Rhizobium etli* CFN42 and its derivative strains were cultured at 28 °C in peptone–yeast (PY) medium [18]. *Agrobacterium tumefaciens* KYC55(PJZ372)(PJZ384)(PJZ410), used for AHL bioassays, was grown at 28 °C in LB or AT minimal medium [19]. For solid media, 1.2% (*w*/*v*) agar was added. When required, antibiotics were added at the following concentrations: streptomycin (Str, 100 μg/mL); spectinomycin (Spe, 50 μg/mL); rifampicin (Rif, 5 μg/mL); kanamycin (Kan, 50 μg/mL); gentamicin (Gen, 5 μg/mL); tetracycline (Tet, 10 μg/mL); chloramphenicol (Chl, 20 μg/mL). Strains were grown to early logarithmic (OD_600_ = 0.2) or stationary (OD_600_ = 1.0) phase as indicated. Bacterial growth was determined by measuring OD_600_ using a spectrophotometer (Philes Ltd., Nanjing, China). Bacterial strains and plasmids used in this study are listed in Table S1.

### 2.2. Transcriptomic Analysis

Total RNA was extracted from nodules (21 days post-inoculation) using TRIzol^®^ Reagent according to the manufacturer’s instructions (Invitrogen, Carlsbad, CA, USA) and genomic DNA was removed using DNase I (TaKara, Tokyo, Japan). Ribosomal RNA was removed, and strand-specific libraries were prepared for paired-end sequencing on an Illumina platform. Total RNA was extracted from the tissue using TRIzol^®^ Reagent according to the manufacturer’s instructions (Invitrogen), and genomic DNA was removed using DNase I (TaKara). Then RNA quality was determined using a 2100 Bioanalyser (Agilent, Santa Clara, CA, USA) and quantified using an ND-2000 (NanoDrop Technologies, Wilmington, DE, USA). A high-quality RNA sample (OD260/280 = 1.8~2.2, OD260/230 ≥ 2.0, RIN ≥ 6.5, 28S:18S ≥ 1.0, >10 μg) was used to construct the sequencing library. RNA-seq strand-specific libraries were prepared with a TruSeq RNA sample preparation Kit from Illumina (San Diego, CA, USA), using 5 μg of total RNA. Shortly, rRNA removal was conducted using a RiboZero rRNA removal kit (Epicenter, Madison, WI, USA) and fragmented using a fragmentation buffer. cDNA synthesis, end repair, A-base addition and ligation of the Illumina-indexed adaptors were performed according to Illumina’s protocol. Libraries were then size-selected for cDNA target fragments of 200–300 bp on 2% Low-Range Ultra Agarose, followed by PCR amplification using Phusion DNA polymerase (NEB) for 15 PCR cycles. After quantification by TBS380, paired-end libraries were sequenced by Illumina NovaSeq 6000 sequencing (150 bp×2, Shanghai BIOZERON Co., Ltd., Shanghai, China). The raw paired-end reads were trimmed and quality controlled by Trimmomatic with parameters (SLIDINGWINDOW:4:15 MINLEN:75) (version 0.36 http://www.usadellab.org/cms/uploads/supplementary/Trimmomatic) (accessed on 3 May 2025). Then clean reads were separately aligned to the reference genome with orientation mode using Rockhopper (version 2.0.3 http://cs.wellesley.edu/~btjaden/Rockhopper/) (accessed on 6 May 2025) software. Rockhopper is a comprehensive and user-friendly system for computational analysis of bacterial RNA-seq data. As input, Rockhopper takes RNA sequencing reads generated by high-throughput sequencing technology. This software was used to calculate gene expression levels with default parameters. Reads were mapped to the *R. etli* CFN42 and *P. vulgaris* reference genomes. Three biological replicates per condition were sequenced. To identify DEGs (differentially expressed genes) between the two different samples, the expression level for each transcript was calculated using the fragments per kilobase of read per million mapped reads (RPKM) method. EdgeR (version 4.6.2 https://bioconductor.org/packages/release/bioc/html/edgeR.html) (accessed on 13 May 2026) was used for differential expression analysis. The DEGs between the two samples were selected using the following criteria: (i) the logarithm of fold change was greater than 2, and the false discovery rate (FDR) was less than 0.05. To understand the functions of the differentially expressed genes, GO functional enrichment and KEGG pathway analysis were carried out by Goatools (version 1.4.11 https://github.com/tanghaibao/Goatools) (accessed on 15 May 2026) and KOBAS (version.3.0 http://kobas.cbi.pku.edu.cn/home.do) (accessed on 15 May 2026), respectively. DEGs were significantly enriched in GO terms and metabolic pathways when their Bonferroni-corrected *p*-value was less than 0.05.

### 2.3. Construction of In-Frame Deletion and Complementation

*R.etli* CFN42 was used as the parental strain for generating the in-frame deletion of *oxyR* (RHE_RS23860) following a previously described method [16]. Briefly, the flanking fragments of the gene *oxyR* were cloned into a suicide vector pEX18Gm containing the *sacB* gene. Table S2 lists the primers used to generate upstream and downstream regions. For complementation analysis, the coding regions of *cinR* and *katG* were amplified and cloned into the plasmid pYC12 by PCR using the primers listed in Table S2 [20]. The resulting recombination plasmids were introduced into the corresponding deletion mutant strain by electroporation.

### 2.4. Disc Diffusion Assay

Bacterial cultures grown to early-log phase or stationary phase were adjusted to 10^9^ bacterial cells per mL. The suspension was mixed with semi-solid PY medium (0.6% agar) and poured over a base of PY medium (1.2% agar). Sterile filter paper discs (6 mm diameter) were placed on the surface and 4 μL of 10 M H_2_O_2_, 4 μL of 4.88 M CuOOH, or 1 μL of 6.46 M tBOOH was applied. After 48 h of incubation at 28 °C, the diameter of the inhibition zone was measured. All assays were performed in triplicate.

### 2.5. Peroxide Killing Assay

Cells were harvested from early-log or stationary phase cultures, washed twice, and resuspended in PBS (pH 7.4) to a density of 10^9^ bacterial cells per mL. The suspensions were aliquoted into a 24-well plate and treated with various concentrations of H_2_O_2_ (0–10 mM). After 2 h of incubation at 28 °C with gentle shaking, samples were serially diluted in PBS and spotted on PY agar plates. Colonies were counted after 3 days of incubation at 28 °C to determine CFU/mL. Survival was calculated relative to the untreated control. Each experiment was performed with three biological replicates.

### 2.6. β-Galactosidase Activity Assays

For strains carrying pRA302-based translational fusions, cells were grown to the desired OD_600_, with or without H_2_O_2_ induction for 30 min [21]. β-galactosidase activity was measured as described by Miller, using cells permeabilized with SDS and chloroform. Activity of *R. etli* strains was expressed in Miller Units, as described previously [8]. All assays were performed with at least three biological replicates.

### 2.7. AHL Extraction and Bioassay

AHLs were extracted from stationary phase culture supernatants of *R. etli* CFN42 using acidified ethyl acetate. The organic phase was dried by rotary evaporation, and the residue was resuspended in 1 mL of ethyl acetate [8]. For bioassays, the extract was added to the AHL biosensor strain *A. tumefaciens* KYC55(PJZ372)(PJZ384)(PJZ410) growing in AT medium [19]. β-galactosidase activity from the biosensor’s *lacZ* reporter was measured as described above to quantify relative AHL levels. Briefly, the AHL biosensor strain was inoculated into AT medium at a ratio of 1:100, and the rhizobium culture supernatants collected at different time points were added separately. The cells were cultured to an OD_600_ of 0.2~1.0. In a 2 mL centrifuge tube, Z-buffer, 0.5% SDS, CHCl_3_, and 0.2 mL of the above bacterial culture were mixed and vortexed thoroughly. The chromogenic substrate ONPG was added, and the reaction was timed until the solution turned yellow. Then, 1 M Na_2_CO_3_ was added to terminate the reaction [8].

### 2.8. Bacterial One-Hybrid (B1H) Assay

The *E. coli* XL1-Blue MRF’ Kan strain was used for the routine propagation of all pBXcmT and pTRG recombinant plasmids [22]. The coding region of *cinR* was cloned into the target vector pTRG. The *katG* promoter region was cloned into the bait vector pBXcmT. A pair of pBXcmT/pTRG plasmids was co-transformed into the reporter strain, and its growth was then tested, together with the self-activation control, on a selective medium containing 3-AT, Kan, Str, and Chl. Positive co-transformants were selected on selective screening medium plates containing 20 mM-AT, 16 μg/mL streptomycin, 15 μg/mL tetracycline, 34 μg/mL chloramphenicol, and 50 μg/mL kanamycin. The plates were incubated at 30 °C for 3–4 d.

### 2.9. Electrophoretic Mobility Shift Assay (EMSA)

His-tagged CinR protein was overexpressed in *E. coli* BL21 (DE3) and purified using Ni-NTA affinity chromatography [8]. DNA probes corresponding to the *katG* promoter region were generated by PCR and purified. Binding reactions were carried out in a 15 μL volume containing binding buffer (50 mM Tris-HCl (pH 8.3), 0.25 M KCl, 2.5 mM DTT, 5 mM MgCl_2_, 0.05 μg/mL poly(dI-dC), 2.5 mM EDTA, 1% glycerol), 50 ng of probe, and increasing amounts of purified CinR protein. Where indicated, 1 μL of AHL extract was added. After incubation at 4 °C for 20 min, samples were loaded onto a 6% native polyacrylamide gel in 0.5 × Tris–borate–EDTA buffer at 150 V for 70 min. The gel was subsequently stained with GelRed (Sangon Biotech, Shanghai, China) for 20 min and then imaged while using the gel imaging system.

### 2.10. Plant Nodulation Assays and Nitrogenase Activity

Common bean (*Phaseolus vulgaris* L.) seeds were surface-sterilized in 2% NaClO for 3 min and subsequently rinsed 7~8 times with sterile distilled water. Sterilized seeds were germinated on 1% agar powder plates prepared with distilled water and incubated in the dark at 28 °C for 2–3 days. Sprouted seeds (root length about 1.5 cm) were transferred to sterile growth tubes containing nitrogen-free nutrient solution (0.132 g/L CaCl_2_, 0.12 g/L MgSO_4_∙7H_2_O, 0.1 g/L KH_2_PO_4_, 0.075 g/L Na_2_HPO_4_∙2H_2_O, 5 mg/L Fe-citrate, and 0.07 mg/L each of MnCl_2_∙4H_2_O, CuSO_4_∙5H_2_O, ZnCl_2_, H_3_BO_3_, and Na_2_MoO_4_∙2H_2_O, adjusted to pH 7.5 before autoclaving) [23]. For inoculation, bacterial cultures were washed and resuspended in sterile water to 10^9^ CFU/mL. The roots of sprouted seeds were treated using a pipette with 100 μL of bacterial suspension. Plants were grown in a controlled environment chamber (28 °C, 16/8 h light/dark cycle) at 5 days post-inoculation (dpi). Nodules were harvested at indicated time points for downstream analyses. The nitrogenase activity was assessed by the acetylene reduction assay (ARA test), as reported previously [24]. Fresh nodules were incubated in sealed vials containing 10% (*v*/*v*) acetylene for 2 h at 28 °C. Ethylene production was quantified using a gas chromatograph (Agilent 7890B) equipped with a flame ionization detector and a Porapak N column. Activity was expressed as μmol C_2_H_4_ produced h^−1^ g^−1^ nodule dry weight [25,26].

### 2.11. Histochemical Staining of Nodule Sections

Fresh nodules were embedded in Tissue-Tek O.C.T. compound and sectioned (50 μm thickness) using a cryostat. For H_2_O_2_ detection, sections were stained with 1 mg/mL DAB (3,3′-diaminobenzidine) solution in PBS (pH 7.4) for 30 min in the dark [27]. For O_2_^•−^ detection, sections were stained with 0.5 mg/mL NBT (nitroblue tetrazolium) solution in PBS for 30 min in the dark [28]. After staining, sections showing formazan blue precipitates (reaction between NBT and O_2_^•−^) or a reddish-brown derivative (reaction between DAB and H_2_O_2_) were washed with distilled water and observed under a light microscope (Olympus BX53).

### 2.12. Statistical Analysis

All experiments were performed with at least three independent biological replicates. Data are presented as mean ± standard error of the mean (SEM). Statistical significance between two groups was determined by unpaired two-tailed Student’s *t*-test. For comparisons among multiple groups, one-way analysis of variance (ANOVA) followed by Tukey’s honestly significant difference (HSD) post hoc test was used. A *p*-value < 0.05 was considered statistically significant. All analyses were performed using GraphPad Prism software (version 9).

## 3. Results

### 3.1. Transcriptomic Analysis Revealed That CinR Affected Antioxidant Gene Expression

**Figure 1:**
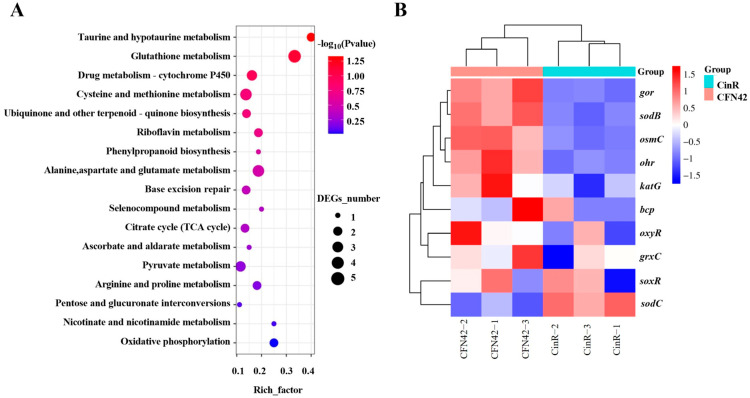
Transcriptomic analysis reveals a role of CinR in oxidative stress defense. (**A**) KEGG pathway enrichment analysis of downregulated genes in Δ*cinR*. (**B**) Heatmap showing the normalized expression levels (RPKM) of selected ROS-related genes in three biological replicates of CFN42 and the Δ*cinR* mutant. CFN42 was used as the control for comparison.

No previous work has reported the regulatory pathway of CinR in *Rhizobium etli* CFN42. Here, we performed transcriptome profiling of CFN42 wild-type and Δ*cinR* mutant strains during their interaction with common bean. A total of 642 differentially expressed genes (DEGs) were identified with the thresholds of |log_2_FC| ≥ 1 and FDR ≤ 0.05. Among these DEGs, 442 genes were upregulated and 200 genes were downregulated in the Δ*cinR* mutant relative to CFN42. The KEGG pathway enrichment analysis revealed that these genes are involved in processes such as organic compound metabolism, bacterial secretion systems, RNA degradation, and protein export. Among the downregulated genes, KEGG pathway enrichment analysis revealed that genes associated with environmental stress resistance, such as glutathione metabolism and drug metabolism, were differentially expressed between CFN42 and the Δ*cinR* mutant (Figure 1A). Most of those metabolic pathways were related to redox responses. Further analysis of genes related to ROS scavenging showed that those genes were significantly downregulated in the Δ*cinR* mutant (Figure 1B), such as *katG* (RHE_PF00004), encoding a functional catalase–peroxidase (log_2_FC = −0.6); *oxyR* (RHE_PF00003), a LysR-type transcriptional regulator of oxidative stress genes (log_2_FC = −1.3); *ohr* (RHE_CH02544), encoding an organic hydroperoxide resistance protein (log_2_FC = −2.2); and *sodB* (RHE_CH01203), encoding an iron superoxide dismutase (log_2_FC = −2.1). This transcriptional profile data strongly indicated that the Δ*cinR* mutant may be deficient in its capacity to control oxidative stress resistance. The heatmap displays RPKM values from individual replicates, with the three CFN42 columns serving as the baseline control for evaluating expression changes in the Δ*cinR* mutant.

### 3.2. Quorum Sensing Regulator CinR Involved in Cell-Density-Dependent H_2_O_2_-Specific Sensitivity in R. etli CFN42

**Figure 2:**
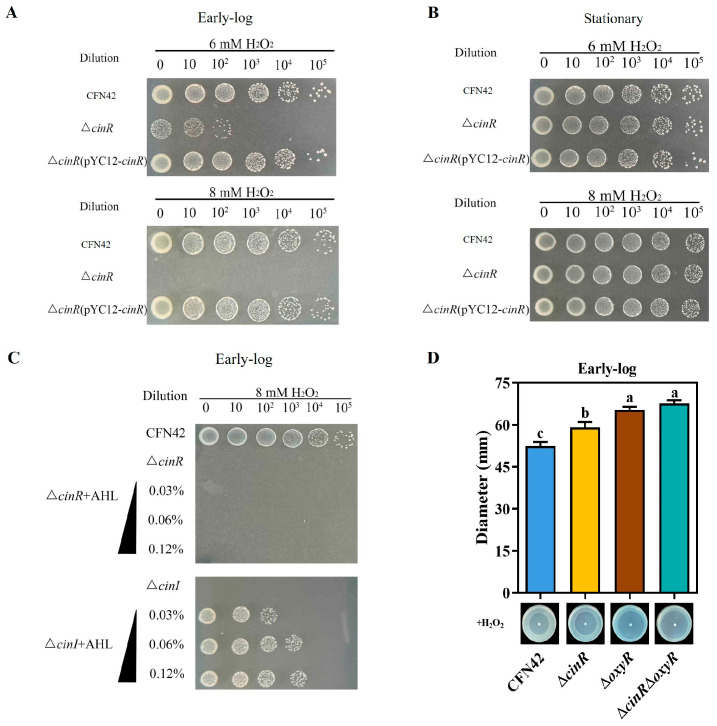
H_2_O_2_ sensitivity assays reveal a role of CinR in oxidative stress defense during early-log phase. (**A**) H_2_O_2_ killing assay for CFN42 and Δ*cinR* at early-log phase (OD_600_ = 0.2). (**B**) H_2_O_2_ killing assay for CFN42 and Δ*cinR* at stationary phase (OD_600_ = 1.0). (**C**) H_2_O_2_ killing assay comparing Δ*cinR* and Δ*cinI* with increasing concentrations (0%, 0.03%, 0.06%, 0.12% *v*/*v*) of exogenously added AHL extract at early-log phase. (**D**) Disc diffusion assay for CFN42, Δ*cinR*, Δ*oxyR* and Δ*cinR*Δ*oxyR* against H_2_O_2_ at early-log phase (OD_600_ = 0.2). Data are mean ± SD (n = 3). Different letters above the columns indicate significant differences by one-way ANOVA (*p* < 0.05).

The transcriptome profiling data indicated that CinR may be involved in ROS resistance in CFN42. We then tested H_2_O_2_ sensitivity and found that the Δ*cinR* mutant strain was more sensitive than the CFN42 strain at the early-log phase, and this phenotype was successfully complemented (Figure 2A and Figure S1A,B). In contrast, no difference in H_2_O_2_ sensitivity between CFN42 and the Δ*cinR* mutant was observed at the stationary phase (Figure 2B). These data indicated that a CinR-mediated cell-density-dependent H_2_O_2_ resistance pathway exists in CFN42. Our previous work revealed that CinR exhibits AHL-independent activity binding to the *cinI* promoter [8]. We then detected H_2_O_2_ sensitivity of the Δ*cinI* mutant under conditions with or without AHLs. In order to determine the concentration of exogenous AHLs, we detected AHL production in the WT, Δ*cinR* and Δ*cinI* strains. We found that adding 0.4% extracted supernatants of *R. etli* CFN42 cultured at the stationary stage is close to the AHL concentration in supernatants of CFN42 at the stationary stage (Figure S2). The results showed that exogenous AHLs restored the H_2_O_2_ sensitivity defect of the Δ*cinI* mutant in a concentration-dependent manner. But exogenous AHLs could not rescue the H_2_O_2_ sensitivity defect of the Δ*cinR* mutant (Figure 2C). These data revealed that the CinR-mediated ROS resistance is an AHL-dependent process. Because CFN42 possesses three QS regulators (CinR, RaiR and TraR), we compared the H_2_O_2_ sensitivity of the three mutants (Δ*cinR*, Δ*raiR* and Δ*traR*) to confirm which regulator was the key in ROS responses. There was no difference among the CFN42, Δ*raiR* and Δ*traR* strains. The result showed that only CinR responds to ROS (Figure S1C). Because the transcriptome profiling data showed that *ohr* was downregulated in the Δ*cinR* mutant, a disc diffusion assay was employed to evaluate sensitivity to organic peroxides, including cumene hydroperoxide (CuOOH) and tert-butyl hydroperoxide (tBOOH), in the three regulator mutants at the early-log phase. These results showed that none of these three regulators was involved in organic peroxide resistance (Figure S1D,E). Because OxyR is the principal regulator that responds to inorganic ROS in bacteria, we constructed a Δ*cinR*Δ*oxyR* double mutant strain and each single-gene mutant (Δ*cinR* and Δ*oxyR*) strain to compare the ROS scavenging capabilities of CinR and OxyR in CFN42. The results showed that OxyR contributed more to H_2_O_2_ resistance than CinR did at the early-log phase (Figure 2D). This pattern was conserved at the stationary phase, while CinR lost regulatory function to ROS resistance (Figure S1F). All these data indicated that, unlike in most other bacteria, CinR in *R. etli* CFN42 plays a critical role in inorganic ROS resistance at the early-log phase in an AHLs dose-dependent regulatory process.

### 3.3. CinR Directly Regulates katG Expression Depending on AHLs

**Figure 3:**
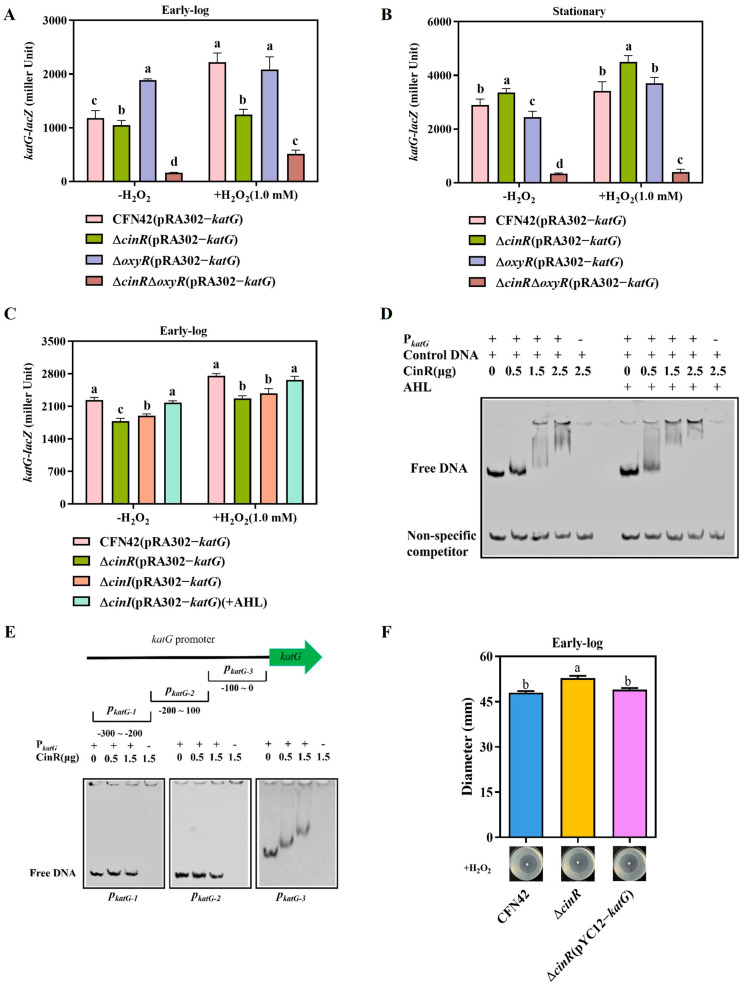
CinR directly binds the *katG* promoter to activate its expression in an AHL-enhanced manner. (**A**) β-galactosidase activity from P*_katG_*-*lacZ* translational fusion in CFN42, Δ*cinR*, Δ*oxyR*, and Δ*cinR*Δ*oxyR* at early-log phase. (**B**) β-galactosidase activity from P*_katG_*-*lacZ* translational fusion in CFN42, Δ*cinR*, Δ*oxyR*, and Δ*cinR*Δ*oxyR* at stationary phase. (**C**) β-galactosidase activity from P*_katG_*-*lacZ* translational fusion in CFN42, Δ*cinR*, Δ*cinI*, and Δ*cinI* supplemented with exogenous AHL extract (0.12% *v*/*v*) at early-log phase. (**D**) EMSA showing binding of purified CinR protein to the *katG* promoter probe with/without AHL. (**E**) EMSA mapping the CinR binding region using truncated promoter probes P_*katG*-1_ (−300~−200), P_*katG*-2_ (−200~−100), and P_*katG*-3_ (−100~0). (**F**) H_2_O_2_ disc diffusion assay for CFN42, Δ*cinR* and Δ*cinR* (pYC12-*katG*). Data are mean ± SD (n = 3). Different letters above the columns indicate significant differences by one-way ANOVA (*p* < 0.05).

To identify genes regulated by CinR, we performed a transcriptional fusion reporter assay of ROS scavenging genes (*oxyR*, *ohr* and *soxR*) that were downregulated in the Δ*cinR* mutant based on RNA-seq analysis (Figure 1B). The data showed that CinR did not regulate the expression of these genes under conditions with or without oxidative stress (Figure S3A–C). Because *R. etli* CFN42 possesses only one catalase gene (*katG*), we studied its regulation by constructing a *katG*-*lacZ* transcriptional fusion reporter to measure *katG* expression in CFN42, Δ*cinR*, Δ*oxyR* and Δ*cinR*Δ*oxyR* strains under different H_2_O_2_ concentrations [29]. The results showed that CinR positively regulated *katG* expression at the early-log phase under conditions with or without H_2_O_2_, while OxyR showed a weaker effect on *katG* expression (Figure 3A). This pattern was reversed at the stationary phase (Figure 3B). The expression of *katG* was lowest in the Δ*cinR*Δ*oxyR* double mutant strain among these strains under conditions with or without oxidative stress. The expression of *katG* was restored while CinR was complemented into the Δ*cinR* mutant (Figure S3D). The difference between the *katG* expression and disc diffusion assay of the Δ*cinR* mutant and Δ*oxyR* mutant may be due to OxyR, which is a global ROS regulator that not only induces *katG* expression but also induces other ROS scavenging genes (*ahpCD*, *soxR*). Then, the Δ*oxyR* mutant showed more sensitivity to H_2_O_2_ than the Δ*cinR* mutant at the early-log phase. We also detected *katG* expression in the Δ*cinI* mutant and compared it with that in the Δ*cinR* mutant. The result showed that the Δ*cinI* mutant had similar levels of *katG* expression to the Δ*cinR* mutant, and the addition of AHLs restored *katG* expression to near CFN42 levels under conditions with or without H_2_O_2_ (Figure 3C).

To validate and expand upon these results, we used electrophoretic mobility shift assays (EMSAs), which revealed that CinR bound to the *katG* promoter regions. The addition of AHLs increased the binding affinity of CinR for the *katG* promoter (Figure 3D). The result of the bacterial one-hybrid (B1H) assay also confirmed that CinR directly bound to the *katG* promoter (Figure S3F). To map the CinR binding site within the *katG* promoter, we designed three different promoter fragments: P_*katG*-1_, P_*katG*-2_, and P_*katG*-3_. EMSAs revealed that CinR binds to P_*katG*-3_ (Figure 3E). These data indicated that CinR binds the *katG* promoter between the −100 bp and 0 bp region, promoting its transcription in an AHL-dependent manner.

To determine the regulatory function of CinR in *katG* expression, we introduced a *katG* complementary plasmid into the Δ*cinR* mutant. Disc diffusion data showed that the complemented Δ*cinR*(pYC12-*katG*) strain restored ROS resistance to levels similar to those of CFN42 at the early-log phase (Figure 3F). The H_2_O_2_ killing assay also confirmed this result (Figure S3E). The expanded ROS resistance functions of CinR in CFN42 may be related to overcoming ROS triggered by rhizobial infection and energy metabolism during nitrogen fixation.

### 3.4. CinR-katG Regulation Pathway Functions in H_2_O_2_ Scavenging and Promotes Optimal Symbiotic Nitrogen Fixation in Planta

**Figure 4:**
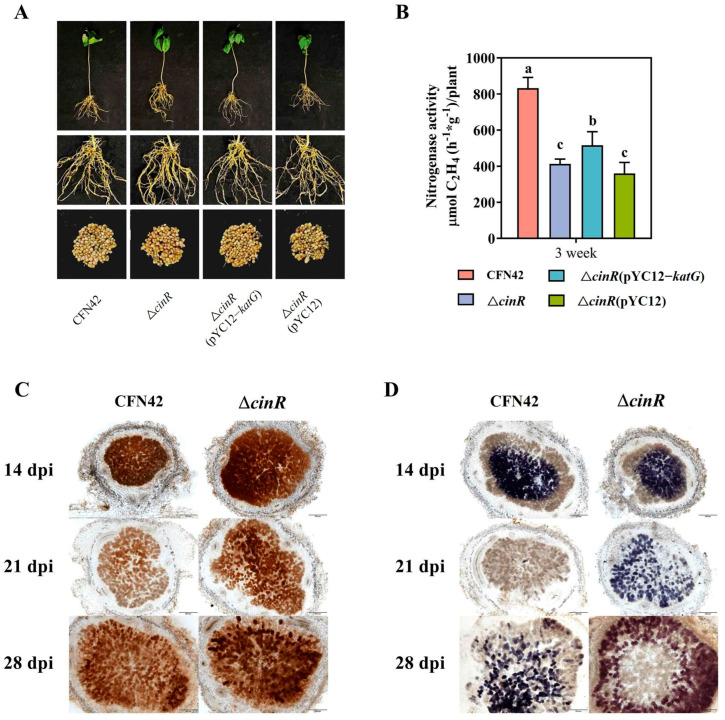
The CinR-*katG* pathway functions *in planta* to limit H_2_O_2_ accumulation and support optimal symbiotic nitrogen fixation. (**A**) Representative images of 3 wpi common bean plants inoculated with the indicated strains. (**B**) Nitrogenase activity (acetylene reduction) of nodules at 3 wpi. (**C**) DAB staining for H_2_O_2_ on nodule sections from 2, 3 and 4 wpi. (**D**) NBT staining for O_2_^•−^ on nodule sections from 2, 3 and 4 wpi. Scale bar = 200 μm. (**C**,**D**) Different letters above the columns indicate significant differences by one-way ANOVA (*p* < 0.05).

To determine the physiological function of the CinR-*katG* regulatory pathway in vivo, we analyzed the symbiotic performance of CFN42, the Δ*cinR* mutant, the complemented Δ*cinR* (pYC12-*katG*) strain, and the vector control Δ*cinR* (pYC12) strain. There was no difference in nodulation ability or growth parameters among these strains during the whole growth period (Figure 4A and Figure S4).

However, nitrogenase activities differed among these strains on 21 dpi. CFN42 root nodules had the highest nitrogenase activity among these strains. The complemented Δ*cinR* (pYC12-*katG*) nodules showed higher nitrogenase activity than those of the Δ*cinR* mutant and Δ*cinR* (pYC12) strains, but lower than that of CFN42 (Figure 4B). The results indicated that *katG* partially restored the nitrogen fixation defect of the Δ*cinR* mutant. This implies that other regulatory pathways of CinR may affect SNF of CFN42.

To determine whether CinR contributes to oxidative stress resistance in vivo, we used nitro-blue tetrazolium (NBT) and 3,3′-diaminobenzidine (DAB) staining to assess the levels of O_2_^•−^ and H_2_O_2_ in the root nodules formed by the CFN42 and Δ*cinR* mutant strains. The results showed that the Δ*cinR* mutant nodules contained higher O_2_^•−^ and H_2_O_2_ concentrations than CFN42 nodules did (Figure 4C,D). The Δ*cinR* mutant nodules showed higher H_2_O_2_ concentration than that of the CFN42 nodules, and the Δ*cinR* (pYC12-*katG*) complementary strain can rescue the defect of ROS scavenging in the Δ*cinR* mutant (Figure S4D). These results indicated that the Δ*cinR* mutant nodules had higher ROS stress than that of CFN42 nodules. These results showed that the Δ*cinR* mutant nodules experienced high oxidative stress, which inhibited nitrogenase activity. In summary, CinR functions as a novel oxidative stress resistance regulator that induces the expression of *katG*. This redox regulation pathway creates a suitable low-oxidative-stress environment during rhizobial CFN42 infection and symbiosis in the common bean, thereby promoting nitrogen fixation.

## 4. Discussion

Bacteria must cope with ROS stress generated by electron flux and energy derived from the TCA cycle and respiratory chain. To overcome the damage caused by ROS to living cells, bacteria employ complex ROS scavenging systems. The main inorganic ROS scavenging regulator is OxyR (a LysR family protein), which senses oxidative status via oxidation of cysteine residues to form disulfide bonds. Normally, OxyR regulates inorganic ROS-scavenging-related genes, such as *katG*, *ahpCD*, and *soxR* [30]. Because BNF is a reductive biochemical reaction, nitrogenase is very sensitive to oxygen. *Azotobacter* species employ multiple oxygen/redox sensors to regulate nitrogenase gene expression, such as NifA, the FixLJ two-component system, RegB, and NifL [31]. Most previous works have focused on OxyR-mediated catalase regulation pathways affecting symbiotic nitrogen fixation, but the exquisite redox regulation in rhizobia is still unclear [16,29,32].

In this study, we employed RNA-Seq analysis and found that the rhizobial quorum sensing regulator CinR directly induces expression of the catalase–peroxidase gene *katG*. AHLs enhance the binding affinity of CinR for the *katG* promoter. This regulatory axis is essential for resistance to H_2_O_2_ in vitro during early growth and, more importantly, functions in planta to limit H_2_O_2_ accumulation within nodules, promoting nitrogenase activity.

The direct regulation of *katG* by CinR represents a novel mechanism integrating population density sensing with antioxidant defense. This is distinct from the function of alkyl hydroperoxide reductase (AhpC), which is also essential for oxidative stress resistance and symbiosis in other rhizobia but is not directly controlled by QS [33]. While QS is known to affect various aspects of symbiosis, its direct control of a primary ROS scavenging enzyme has not been previously demonstrated in rhizobia. In contrast, in the zoonotic pathogen *Streptococcus suis*, the GntR transcription factor is phosphorylated by a serine/threonine kinase, which represses the transcription of NADH oxidase, leading to NADH accumulation and enhanced oxidative stress susceptibility, thereby reducing virulence [34]. The downregulation of *katG* in the Δ*cinR* mutant provides a clear mechanistic explanation for how QS enhances SNF. The inability to efficiently clear H_2_O_2_ leads to its accumulation within the nodule, creating a high-oxidative-stress environment that is hostile to nitrogenase activity (Figure 4C). The partial rescue of nitrogenase activity by overexpressing *katG* in the Δ*cinR* background provides strong evidence that the ROS scavenging defect is a direct and significant inhibitor of symbiotic performance (Figure 4B). The incomplete rescue suggests that CinR is a multi-functional regulator affecting SNF, possibly through its regulation of the Rai and Tra systems, which may affect other symbiotic processes such as infection thread progression, bacteroid differentiation, or energy metabolism [4,35]. Moreover, recent evidence suggests that CinR also mediates beneficial interactions with soil commensal rhizobia to further enhance nodulation efficiency [36].

The CinR-*katG* regulatory pathway mirrors the complex regulation network of CinRI [8]. The regulatory activity of CinR in *katG* expression in the absence of AHL (evident in the intermediate phenotype of Δ*cinI*) allows for a basal level of ROS protection at low cell densities when CFN42 infects the host plant in the early stages. As the bacterial population increases, accumulating AHL binds to CinR and enhances its affinity for the *katG* promoter (Figure 3C), then further upregulates *katG* expression.

OxyR exerts its function on *katG* expression at the mid-log phase. The alternating manner by which CinR and OxyR regulate *katG* expression in CFN42 ensures that rhizobia initiate ROS scavenging earlier than other bacteria when low oxidative stress does not induce OxyR activity. This provides a mechanism for fine-tuning antioxidant capacity according to population density. This is particularly relevant during symbiosis, where the bacterial population transitions from a few founder cells in an infection thread to a dense population of thousands of bacteroids within a single nodule cell [37]. Rhizobia require ROS scavenging ability at all times, and the CinR-OxyR alternating regulation model meets this special requirement for ROS resistance as rhizobia form nodules on the host plant.

## 5. Conclusions

Our work identifies CinR as a direct activator of *katG*, broadening understanding of how QS regulatory networks are integrated into stress responses and nitrogen fixation. In other systems, QS has been linked to oxidative stress tolerance, often through indirect means, such as regulating biofilm formation, which can provide a physical barrier against ROS [38,39]. Our findings highlight a novel QS-mediated regulatory model of ROS resistance, in which a QS regulator functions as an early-stage transcriptional factor, binding to the promoter of a core antioxidant gene and activating its expression in response to its cognate signal. This CinR-OxyR alternating regulation ensures that CFN42 forms efficient symbioses on the common bean.

## Data Availability

The original data presented in this study are openly available in the Genome Sequence Archive (GSA) under accession code CRA042483, but are not publicly available due to a two-year embargo period. Requests for access can be directed to the corresponding authors.

## Supplementary Materials

The following supporting information can be downloaded at: https://www.mdpi.com/article/10.3390/antiox15060752/s1. Table S1: Strains and plasmids for this study; Table S2: Primers for this study; Figure S1: CinR is not required for organic peroxide resistance, and complementation restores H_2_O_2_ sensitivity; Figure S2: AHL production profiles of *R. etli* strains; Figure S3: CinR specifically regulates *katG* expression but not *oxyR*, *ohr*, or *soxR* in *R. etli* CFN42, and the *katG*-complemented strain restores H_2_O_2_ resistance; Figure S4: Additional symbiotic phenotypes for plants inoculated with different *R. etli* strains.
