# Supplementary material for: Quorum Sensing Regulator CinR Directly Activates the Catalase–Peroxidase Gene katG to Alleviate Oxidative Stress and Promote Symbiotic Nitrogen Fixation in Rhizobium etli CFN42
Source: Antioxidants (Basel). 2026 Jun 15;15(6):752. doi: 10.3390/antiox15060752 (PMC13295618; doi:10.3390/antiox15060752)
Supplement: Supplementary file 1 [file antioxidants-15-00752-s001.zip › antioxidants-4337847-supplementary.pdf]

Table S1. Strains and plasmids for this study.

Strains and plasmids	Characteristics	Resource
**Strains**		
Rhizobium etli		
CFN42	Derivative of wild type, Sm^R	[18]
Δ*cinR*	Derivative of CFN42 carrying an in-frame deletion of *cinR*	[8]
Δ*raiR*	Derivative of CFN42 carrying an in-frame deletion of *raiR*	[8]
Δ*traR*	Derivative of CFN42 carrying an in-frame deletion of *traR*	[8]
Δ*cinI*	Derivative of CFN42 carrying an in-frame deletion of *cinI*	[8]
Δ*cinR* (pYC12)	Δ*cinR* carrying the expression construct pYC12	This work
Δ*oxyR*	Derivative of CFN42 carrying an in-frame deletion of *oxyR*	This work
Δ*cinR*Δ*oxyR*	Derivative of Δ*cinR* carrying an in-frame deletion of *oxyR*	This work
Δ*cinR* (pYC12-*cinR*)	Δ*cinR* carrying the expression construct pYC12-*cinR*	This work
Δ*cinR* (pYC12-*katG*)	Δ*cinR* carrying the expression construct pYC12-*katG*	This work
CFN42 (pRA302-*katG*)	CFN42 harboring the translational fusion plasmid pRA302-*katG*	This work
Δ*cinR* (pRA302-*katG*)	Δ*cinR* harboring the translational fusion plasmid pRA302-*katG*	This work
Δ*oxyR* (pRA302-*katG*)	Δ*oxyR* harboring the translational fusion plasmid pRA302-*katG*	This work
Δ*cinR*Δ*oxyR* (pRA302-*katG*)	Δ*cinR*Δ*oxyR* harboring the translational fusion plasmid pRA302-*katG*	This work
CFN42 (pRA302-*oxyR*)	CFN42 harboring the translational fusion plasmid pRA302-*oxyR*	This work
Δ*cinR* (pRA302-*oxyR*)	Δ*cinR* harboring the translational fusion plasmid pRA302-*oxyR*	This work
CFN42 (pRA302-*soxR*)	CFN42 harboring the translational fusion plasmid pRA302-*soxR*	This work
Δ*cinR* (pRA302-*soxR*)	Δ*cinR* harboring the translational fusion plasmid pRA302-*soxR*	This work
CFN42 (pRA302-*ohr*)	CFN42 harboring the translational fusion plasmid pRA302-*ohr*	This work
Δ*cinR* (pRA302-*ohr*)	Δ*cinR* harboring the translational fusion plasmid pRA302-*ohr*	This work
Escherichia coli		
DH5α	Host for cloning	[8]
BL21 (DE3) (pET28a-cinR)	Host for protein expression	[8]
XL1-Blue MRF′ Kan	Host strain for bacterial one-hybrid assay	[22]
XL1-Blue MRF′ Kan (pTRG-*cinR*) (pBT-*katG*)	XL1-Blue harboring the protein expression plasmid pTRG-*cinR* and the DNA cloning plasmid pBT-*katG*	This work
Agrobacterium tumefaciens		
JZA1	AHLs biosensor strain<br>KYC55 (pJZ372) (pJZ384) (pJZ410) (Tc^R, Spe^R, Gm^R)	[19]
**Plasmids**		
pYC12	In trans expression vector, GmR	[20]

pYC12-*cinR*	pYC12 with the coding region of *cinR*, Gm^R	This work
pYC12-*katG*	pYC12 with the coding region of *katG*, Gm^R	This work
pTRG	Plasmid used for protein expression in bacterial one-hybridization assay, Tc^R	[22]
pBXCmT	Plasmid used for DNA cloning in bacterial one hybridization assay, Chl^R	[22]
pTRG-*cinR*	pTRG cloned with the coding region of *cinR*, Tc^R	This work
pBT-*katG*	pBXcmT cloned with *katG* promoter region, Chl^R	This work
pET28a-cinR	pET28a with the coding region of *cinR*, Kan^R	[8]
pRA302	*lacZ* translational fusion vector, Spe^R	[8]
pRA302-*katG*	pRA302 with *katG* promoter region, Spe^R	This work
pRA302-*oxyR*	pRA302 with *oxyR* promoter region, Spe^R	This work
pRA302-*soxR*	pRA302 with *soxR* promoter region, Spe^R	This work
pRA302-*ohr*	pRA302 with *ohr* promoter region, Spe^R	This work

Table S2. Primers for this study.

Primers	Sequence (5′→3′)	Target gene (Locus Tag)	Refseq	Genebank
For deletion				
pEX18Gm-*oxyR*-1	CGGAATTCACGTAGATGAGACCCATCTG (EcoRI)	*oxyR* (RHE_PF00003)	NC_007766.1	CP000138.1
pEX18Gm-*oxyR*-2	CCGCAAGGGACCGAGCTTATCGCAGCAATC	*oxyR* (RHE_PF00003)	NC_007766.1	CP000138.1
pEX18Gm-*oxyR*-3	ATAAGCTCGGTCCCTTGCGGGAAAGGGTTG	*oxyR* (RHE_PF00003)	NC_007766.1	CP000138.1
pEX18Gm-*oxyR*-4	GCTCTAGATCGGCATGGTGCTCTATGGC (XbaI)	*oxyR* (RHE_PF00003)	NC_007766.1	CP000138.1
For complementation				
pYC12-*cinR*-F	GCGGTACCGGTAAAAGGAGATATACATATG (KpnI)	*cinR* (RHE_CH02915)	NC_007761.1	CP000133.1
pYC12-*cinR*-R	CCAAGCTTTCAGGGATTGATGATGCGCAG (HindIII)	*cinR* (RHE_CH02915)	NC_007761.1	CP000133.1
pYC12-*katG*-F	CGGAATTCATGGACAACCCCACTGACAC (EcoRI)	*katG* (RHE_PF00004)	NC_007766.1	CP000138.1
pYC12-*katG*-R	CCAAGCTTTCAGACGAGGTCGAAGCGGT (HindIII)	*katG* (RHE_PF00004)	NC_007766.1	CP000138.1
For translational fusion				
pRA302-*katG*-F	CGGAATTCCTTTTGTTAAGATCGTTTAT (EcoRI)	*katG* (RHE_PF00004)	NC_007766.1	CP000138.1
pRA302-*katG*-R	CCAAGCTTGATCGTCTCCTTCATTGTTT (HindIII)	*katG* (RHE_PF00004)	NC_007766.1	CP000138.1
pRA302-*oxyR*-F	GCGGTACCACCCTCATCGTATGAGGGACTT (KpnI)	*oxyR* (RHE_PF00003)	NC_007766.1	CP000138.1
pRA302-*oxyR*-R	CCAAGCTTCCAATAAGTTTATCTTATTCAA (HindIII)	*oxyR* (RHE_PF00003)	NC_007766.1	CP000138.1
pRA302-*soxR*-F	CGGAATTCCTGCGGCCGAACCGGCCGCTG (EcoRI)	*soxR* (RHE_CH03863)	NC_007761.1	CP000133.1
pRA302-*soxR*-R	CCAAGCTTCCCGGCAGTGCGATGGCTCAG (HindIII)	*soxR* (RHE_CH03863)	NC_007761.1	CP000133.1
pRA302-*ohr*-F	GCTCTAGACCGGCTGGCACCGCGTCCGT (XbaI)	*ohr* (RHE_CH02544)	NC_007761.1	CP000133.1
pRA302-*ohr*-R	GCGGTACCGGCAAGGGCCGTCTCTGTGAA (KpnI)	*ohr* (RHE_CH02544)	NC_007761.1	CP000133.1
For B1H				
pTRG-*cinR*-F	GGGGTACCGGTAAAAGGAGATATACATA (KpnI)	*cinR* (RHE_CH02915)	NC_007761.1	CP000133.1

pTRG-*cinR*-R	CCAAGCTTTCAGGGATTGATGATGCGCA (HindIII)	*cinR* (RHE_CH02915)	NC_007761.1	CP000133.1
pBT-*katG*-F	GCGGTACCCTCGAAGAGCTCGCTGCCCA (KpnI)	*katG* (RHE_PF00004)	NC_007766.1	CP000138.1
pBT-*katG*-R	GCTCTAGAGATCGTCTCCTTCATTGTTT (XbaI)	*katG* (RHE_PF00004)	NC_007766.1	CP000138.1
For EMSA				
P_{katG}-F [1]	CTCGAAGAGCTCGCTGCCCA	*katG* (RHE_PF00004)	NC_007766.1	CP000138.1
P_{katG}-R [1]	AAACAATGAAGGAGACGATC	*katG* (RHE_PF00004)	NC_007766.1	CP000138.1
$P_{katG\text{-}1}$-F [1]	CTCGAAGAGCTCGCTGCCCA	*katG* (RHE_PF00004)	NC_007766.1	CP000138.1
$P_{katG\text{-}1}$-R [1]	CCGATTTCGCCGTGCCGCCG	*katG* (RHE_PF00004)	NC_007766.1	CP000138.1
$P_{katG\text{-}2}$-F [1]	CCGTCCCTTGCCAAAGCTTC	*katG* (RHE_PF00004)	NC_007766.1	CP000138.1
$P_{katG\text{-}2}$-R [1]	TAATTTTAAGTTGGATTTAC	*katG* (RHE_PF00004)	NC_007766.1	CP000138.1
$P_{katG\text{-}3}$-F [1]	TTGAACGGTTCCGATATGAA	*katG* (RHE_PF00004)	NC_007766.1	CP000138.1
$P_{katG\text{-}3}$-R [1]	GATCGTCTCCTTCATTGTTT	*katG* (RHE_PF00004)	NC_007766.1	CP000138.1
$P_{\text{non-specific competitor}}$-F [1]	CGCCGCTGCTCAAGCGCCTGGAGGG	Intergenic sequence	NC_007761.1	CP000133.1
$P_{\text{non-specific competitor}}$-R [1]	CGTGAGCTGGACGCGCAGGCTTTCC	Intergenic sequence	NC_007761.1	CP000133.1

[1] The prefix "P" indicates the promoter region.

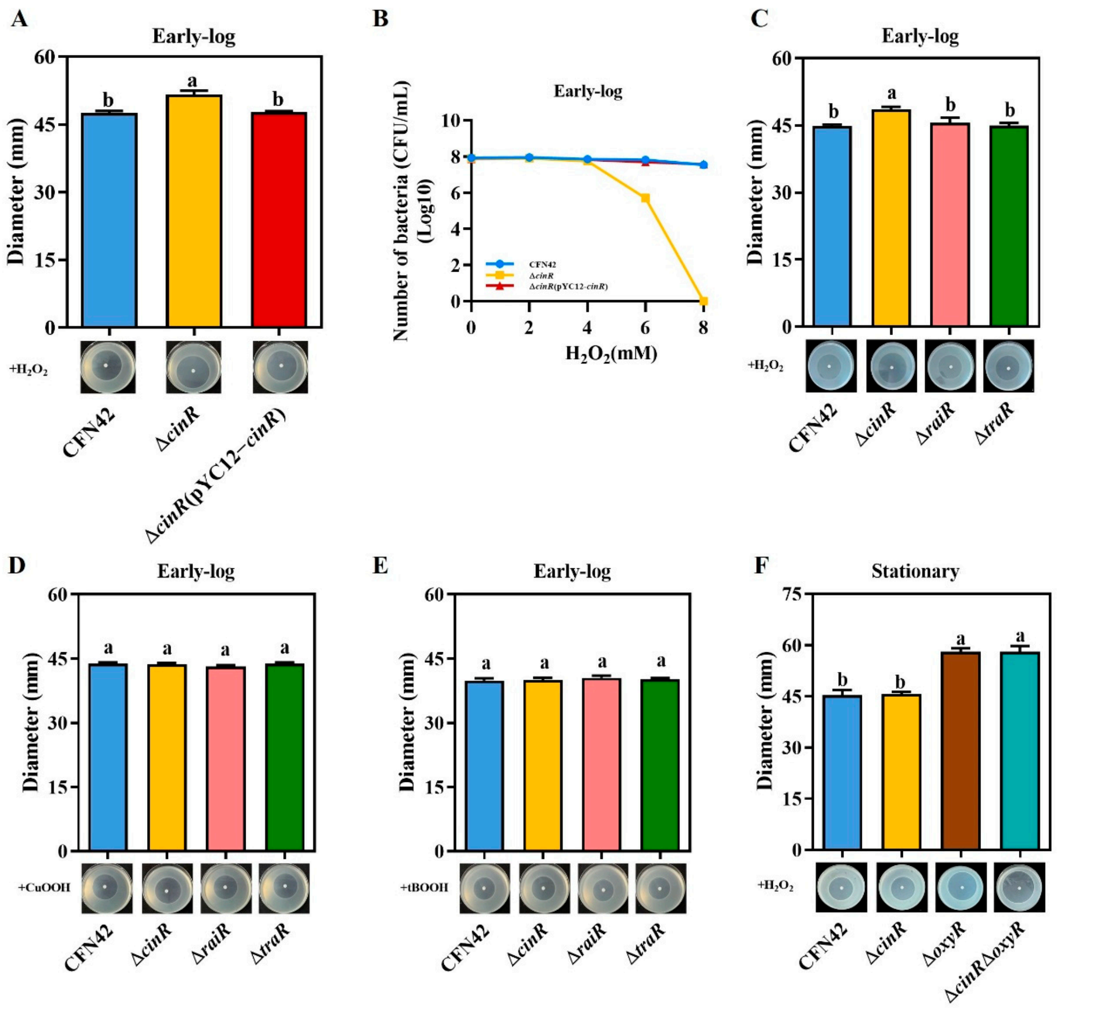

Figure S1. CinR is not required for organic peroxide resistance, and complementation restores H_2O_2 sensitivity. (A) Disc diffusion assay against H_2O_2 for CFN42, Δ*cinR*, and Δ*cinR* (pYC12-*cinR)* at early-log phase, (B) H_2O_2 killing assay for CFN42, Δ*cinR*, and Δ*cinR* (pYC12-*cinR)* at early-log phase, (C-E) Disk diffusion assay for CFN42 and QS mutants against H_2O_2 (C), CuOOH (D) and tBOOH (E) at early-log phase, (F) Disc diffusion assay for CFN42, Δ*cinR*, Δ*oxyR*, and Δ*cinR*Δ*oxyR* at stationary phase. Data are mean ± SD (n = 3). Different letters above the columns indicate significant differences by one-way ANOVA ($p < 0.05$).

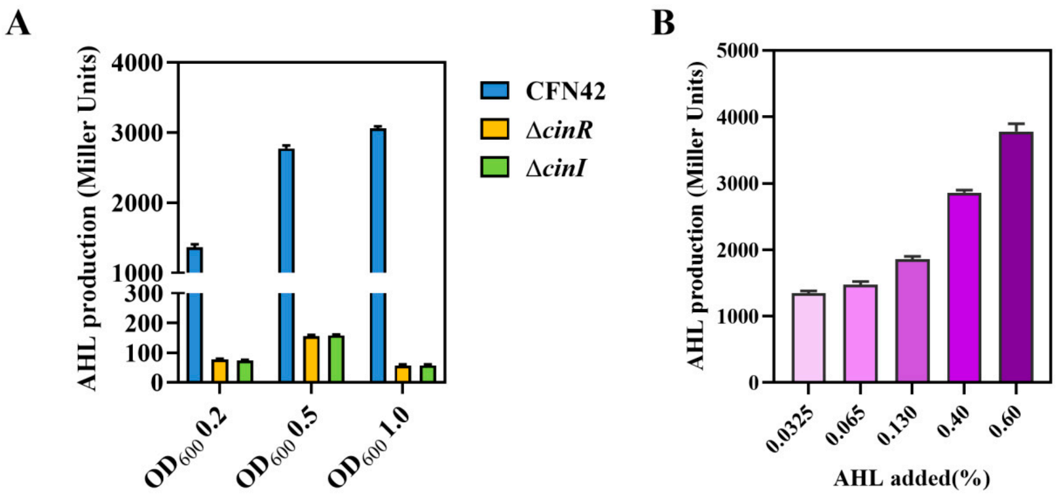

Figure S2. AHL production profiles of *R. etli* strains. (A) AHL production by CFN42, Δ*cinR*, and Δ*cinI* at different growth stages (OD_{600} = 0.2, 0.5 or 1.0), measured using the *A. tumefaciens* JZA1 biosensor. β-galactosidase activity is expressed in Miller Units. (B) Linear determination of AHLs produced by the CFN42 from stationary phase culture. Data are mean ± SD (n = 3).

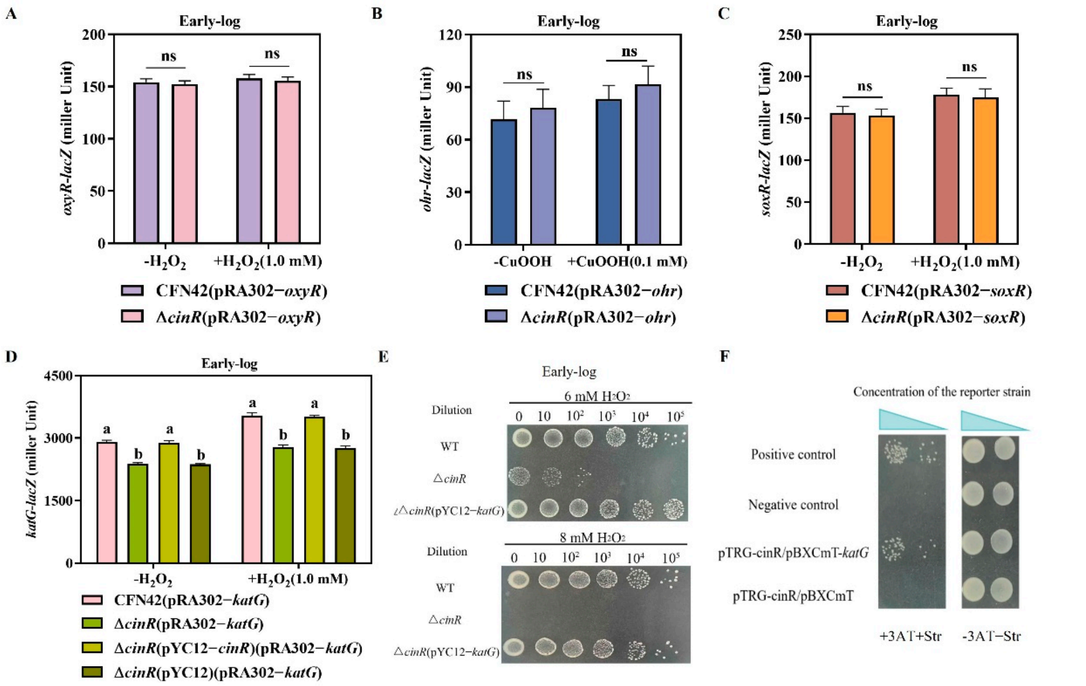

Figure S3. CinR specifically regulates *katG* expression but not *oxyR*, *ohr*, or *soxR* in *R. etli* CFN42, and the *katG*-complemented strain restores H_2O_2 resistance. (A-C) Translational *lacZ* fusion analysis of *oxyR* (A), *ohr* (B), and *soxR* (C) expression in CFN42 and Δ*cinR* at early-log (Data are shown as mean ± SD and reflect the combined results of three independent experiments. Student's t-test, ns: no significant difference), (D) Translational *lacZ* fusion analysis of *katG* expression in CFN42, Δ*cinR*, Δ*cinR* (pYC12-*cinR*), and Δ*cinR* (pYC12) at early-log stage. Different letters above the columns indicate significant differences by one-way ANOVA ($p < 0.05$). (E) H_2O_2 killing assay for CFN42, Δ*cinR* and Δ*cinR* (pYC12-*katG*), (F) Bacterial one-hybrid assay showing interaction between CinR and the *katG* promoter.

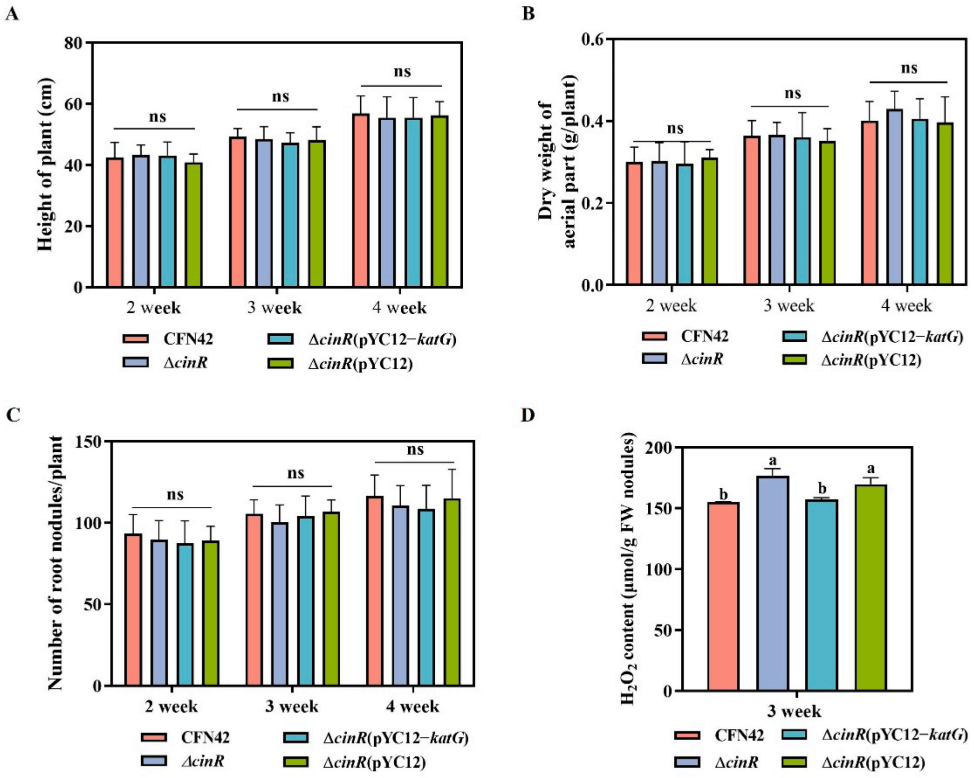

Figure S4. Additional symbiotic phenotypes for plants inoculated with different *R. etli* strains. (A) Height of plants, (B) Dry weight of plants, (C) Number of nodules. Data are mean ± SD (n = 8~10 plants per treatment). (D) H_2O_2 content in root nodules. Different letters above the columns indicate significant differences by one-way ANOVA ($p < 0.05$), ns: no significant difference.
